# The impact of localised general practice training on Queensland’s rural and remote general practice workforce

**DOI:** 10.1186/s12909-020-02025-4

**Published:** 2020-04-20

**Authors:** Raquel Peel, Louise Young, Carole Reeve, Katerina Kanakis, Bunmi Malau-Aduli, Tarun Sen Gupta, Richard Hays

**Affiliations:** 1 College of Medicine and Dentistry, James Cook University, Townsville, Queensland Australia; 2 School of Psychology and Counselling, University of Southern Queensland, Ipswich, Queensland Australia

**Keywords:** GP training, Medical education, Primary care services, Rural and remote health, Remote underserved communities, Medical workforce shortage, Rural general practice, Health care equity, Qualitative research, Thematic analysis

## Abstract

**Background:**

The diverse rural medical education initiatives that have been developed in Australia to address the medical workforce maldistribution have been less successful in many smaller and remote communities. This study explored the factors that attract and retain GP registrars and supervisors and the impact that localised training (i.e., rural and remote workplace-based training and support) has on both GP registrars and supervisors, and the GP workforce in rural and remote underserved areas.

**Methods:**

A purposive sample of 79 GP registrars, supervisors, practice managers, health services staff and community representatives living and working in areas of low GP workforce in rural and remote Australia were invited to participate in semi-structured interviews and one focus group divided over two phases. Thematic analysis was used to explore themes within the data.

**Findings:**

Attractors and barriers to rural and remote practice were identified as the main themes. Attractors include family and community lifestyle factors, individual intrinsic motivators, and remote medicine experiences. In contrast, barriers include work related, location, or family factors. Further, localised GP training was reported to specifically influence GP registrars and supervisors through education, social and financial factors.

**Conclusion:**

The current study has provided a contemporary overview of the issues encountered in expanding GP training capacity in rural and remote communities to improve the alignment of training opportunities with community and workforce needs. Strategies including matching scope of practice to registrar interests have been implemented to promote the attractors and lessen the barriers associated with rural and remote practice.

## Background

Despite over 20 years of rural medical education initiatives in Australia, success in addressing the medical workforce maldistribution remains mixed. While the situation has improved in most regional and coastal rural communities, change has been less evident in many smaller, remote and isolated communities [1]. More is now known about how to recruit rural general practitioners (GPs) by combining a variety of strategies, such as increasing the proportion of rural background students, training them in rurally-oriented medical schools and rural clinical schools, and providing increased specialty training places in regional and rural communities [2–4]. Australia has invested heavily in initiatives during medical school and improving links to specialty training programs. These initiatives increase interest and intention to consider rural careers [5].

However, in the course of a medical career ‘life happens’ (e.g., marriage, children, aging parents) and junior doctors have experiences that may influence career preferences. Some authors argue that personal factors – partners’ work, family and friend support – may be more influential than other factors in deciding where to live and work [6]. Some factors that influence the decision to practise in rural and remote communities include an increased workload; staffing issues; difficulty accessing professional development; and geographical, social and professional isolation [7, 8]. However, rural and remote general practice is regarded as a rewarding career [9]. Previous research has identified that GPs reported the positive aspects to working in rural and remote areas included a wide scope of practice, professional autonomy and the rural lifestyle [10].

Given what is known regarding the barriers and benefits to rural and remote general practice, localised or distributed GP registrar training might simultaneously lessen the barriers and promote the benefits for working in rural and remote areas. Supported learning in smaller communities may assist in combating some of the barriers to working in rural and remote communities. For example, localised training may facilitate the development of a community of practice for both GP registrars and supervisors [11], reducing a sense of professional and social isolation. Knowledge can be shared through the networks of communities of practice both within and across communities, providing support from colleagues who utilise their understanding of the context in framing advice. For instance, research has identified that the provision of professional and social support during internship within underserved areas assists in improving the recruitment of doctors to these underserved areas [11].

**Fig. 1:**
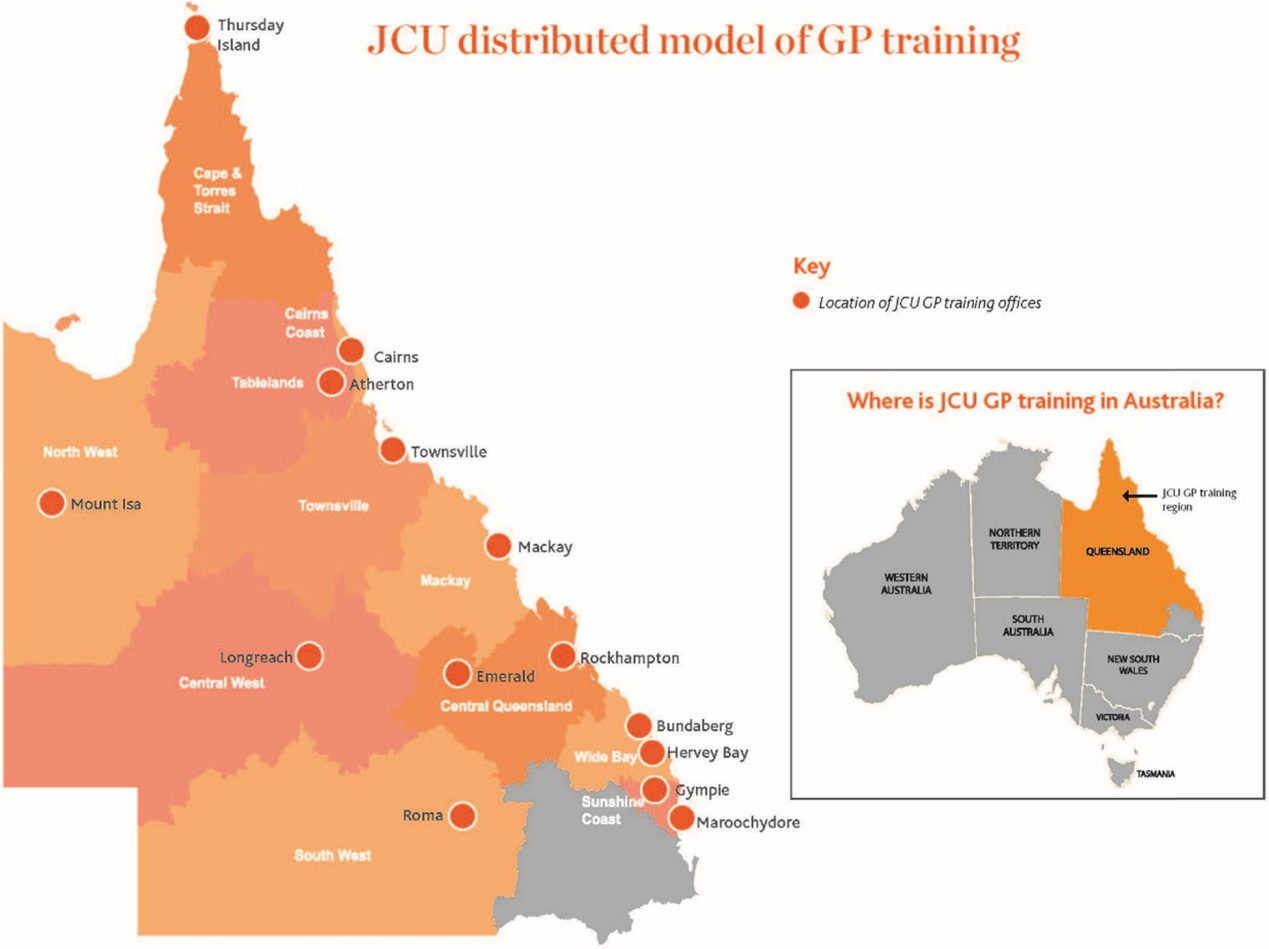
JCU GPT Distributed Model of Training

Much Australian research about recruitment and retention of rural GPs pre-dates the entry to GP training of medical school graduates from the rurally enriched pathways developed over the past two decades [8, 12], and the effects of these supported rural pathways are only just becoming apparent [13, 14]. Previous training models were less engaged with smaller communities in more remote locations, and more focused on larger coastal communities. Therefore, the James Cook University GP Training (JCU GPT) program was implemented in 2016 with a strategy to place and locally train more registrars in smaller rural and remote communities using a distributed model, so-called localised GP training. The JCU GPT localised training incorporates a distributed model that provides workplace based training and increased support from administrative offices in 14 towns throughout central, northern and western Queensland, as shown in Fig. 1.

Prior to 2016, GP training in north western Queensland was provided by one central office. The distributed JCU GPT training model commenced with offices in five larger regional centres (i.e., Atherton, Cairns, Mackay, Mt Isa, and Townsville), where undergraduate training was already established. The model expanded rapidly and now has 11 regional hubs (some with more than one office location) supporting the surrounding small rural towns where GP registrars are placed. In some locations (e.g., Thursday Island, Atherton and Mt Isa) existing JCU GPT structures were strengthened, while in others (e.g., Bundaberg and Emerald) local training nodes were established for the first time. Overall, the localised GP training model ensures individualised support for registrars, whereby each registrar is designated a single training advisor with responsibility to help map their training pathway, review progress at regular intervals, to pro-actively consider the curriculum and assessment issues (for instance, in making best use of hospital and community general practice training time) and in facilitating access to training posts. In addition to their in-situ supervisor and medical educator, each registrar is supported by a designated administration staff member at their regional node. Furthermore, face-to-face and online teaching platforms are provided to facilitate peer-networking and peer-initiated educational engagement.

The aim of the current study was to investigate the attractors and barriers for GP registrars to train and GP supervisors to work in smaller, more remote communities. The current study explored the factors that attract and retain GP registrars and supervisors to rural and remote underserved areas and the impact that localised training has on the GP workforce within rural and remote areas. Localised training was implemented with the expectation that it would contribute to community connectedness. Further, it may also develop a community of practice that provides essential primary care to the community while stabilising the GP workforce.

## Methods

### Design

This was a qualitative study based on a series of semi-structured interviews and one focus group with GP registrars, supervisors, practice managers, health services staff and community representatives. The first phase of interviews explored the perceived attractors and barriers to working and training remotely for GP registrars and supervisors; specifically, it focused on factors influencing recruitment, retention, remote medical practice and GP training. The second phase of interviews and one focus group aimed to provide greater insight into the perceived effect localised training had on GP registrars, supervisors and the community, including the impact on the GP workforce.

### Interview and focus group protocols

The semi-structured interview and focus group protocols for the two phases were developed by all researchers. The questions sought to explore the experiences of GP registrars, supervisors, practice managers, health services staff and community representative living and working in areas of low GP workforce in rural and remote Australia. Specifically, the interview guides included questions regarding attractors, motivators and barriers to rural practice (e.g., What do you enjoy [and not enjoy] about rural/remote practice?), work-life balance and burn-out (e.g., What is important to you when considering work-life balance?), resilience and coping (e.g., How would you describe the particular stressors of living in this community?), supervision (e.g., How are you supported by your supervisor [or how do you support your registrars] with challenges in practice?) and social and economic aspects impacting rural and remote life and work (e.g., What would you say is different about North/West Queensland rural/remote practice?). Focus group questions covered topics such as quality of medical practice training (e.g., Did you choose the town/ practice for training and if so why?), supervision (e.g., What kind of supervision do you most value in a clinical placement?) and community engagement (e.g., How do you contribute to the local community outside of work?).

### Procedure

Interviews were conducted from November 2017 to February 2018 (phase 1) and from November 2018 to March 2019 (phase 2), either face to face or by phone. Two researchers (RP, KK) who conducted the interviews and focus group were employed as research officers for JCU GPT and they did not know the participants prior to the interviews and focus group. Both researchers had experience working as qualitative research officers in other projects and both researchers had conducted PhDs involving qualitative methods. One additional researcher (RH) was involved in conducting the focus group and is a medical educator employed by JCU.

Data was collected until saturation was reached (i.e., additional data did not provide new information) [15]. Interviews lasted between 20 min to 1 h. Interviews were audio recorded and transcribed verbatim. All transcripts were sent to participants for checking and confirmation prior to analysis. Ethics approval was obtained from the JCU Human Research Ethics Committee (Approval Numbers: H7132 and H7497).

### Recruitment

The first phase of interviews recruited a purposive sample of GP registrars, supervisors, and practice managers living and working in areas identified as having low GP workforce in rural and remote Queensland [16]. Invitations were sent out via email to those working in towns in Modified Monash Model (MMM) areas 4–7 (i.e., populations less than 5000 and remote areas; approximately 65 registrars, 45 supervisors and 30 practices) and interested participants replied to the request. Interviews were conducted either by phone or in person.

The second phase, which involved interviews and one focus group, recruited a purposive sample of GP registrars, supervisors, practice managers, health services staff and community representatives living and working in areas of low GP workforce and a low Index of Access (IA) to health services in rural and remote Queensland [16]. Key informants in the underserved communities were contacted via email and invitations for participants sent out through their networks. Invitations to participate were also sent to the relevant federal, state and local level government members, healthcare services and community volunteer groups (e.g., Rotary, Country Women’s Association). Interested participants replied to the request. Participants were from remote, and some rural and regional towns in MMM areas 2–7 (i.e., inner regional, outer regional and remote areas) either by phone or in person. The focus group was held face-to-face with registrars during a training day.

### Data analysis

Interview data was interpreted using thematic analysis with the NVivo 12 software [17]. Analyses were conducted as an iterative process as described by Braun and Clarke [18] – i.e., data familiarisation, generation of initial codes, organisation of potential themes, revision of themes, generation of themes definitions and names, and generation of analysis report. Coding was confirmed using shared coding sessions and theme generation by four researchers (RP, LY, CR, KK) with consensus used to resolve discrepancies. In addition to the two research officers who collected the data, analyses were also conducted by two researchers (LY, CR) who are medical educators, with one (CR) employed by JCU GPT. Final main themes and sub-themes were confirmed by all authors. All findings from the current study were reported in accordance with Tong, Sainsbury, and Craig’s [19] checklist for reporting qualitative interviews.

### Findings

#### Participants

The first phase of interviews recruited a total of 39 participants (i.e., 20 females, 19 males). The second phase of interviews and focus group recruited a total of 40 participants (i.e., 19 females, 21 males). Demographic data for all participants is shown in Table 1.

**Table 1:** Participant demographics

Characteristics		First round	Second round
Age	20–30	6	9
	31–40	12	2
	41–50	14	7
	51–60	7	8
	61–70	0	9
	71–80	0	5
Group	Community Members	0	21
	Supervisor	12	7
	Registrar	14	8
	Practice Manager and Healthcare Services Staff	13	4
Region	Cape York and Torres Strait	2	3
	Central Queensland	0	7
	Central West Queensland	8	17
	North West Queensland	13	7
	South West Queensland	16	3
	Wide Bay	0	3

#### Phase 1 findings

**Fig. 2:**
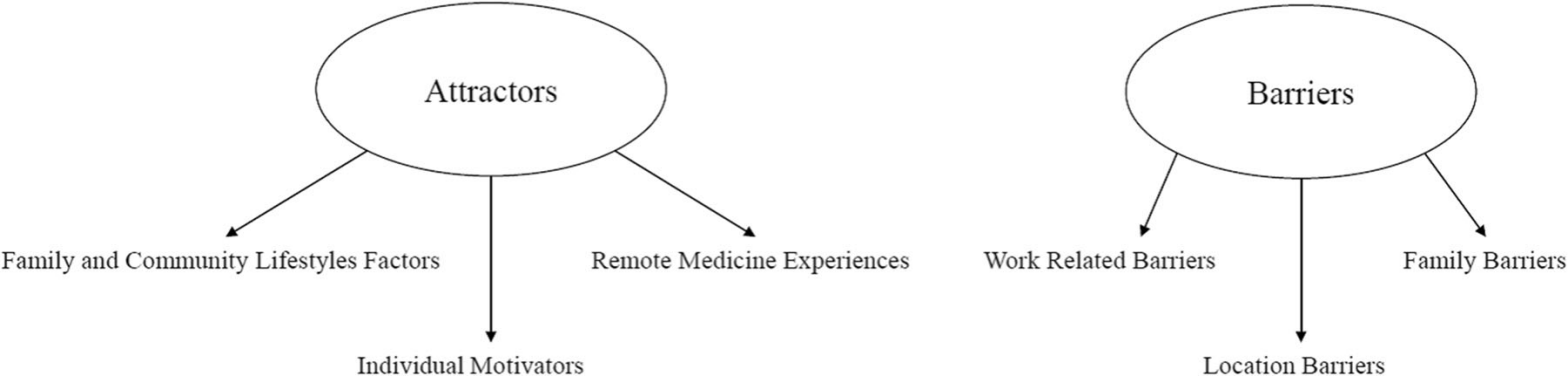
Attractors and Barriers to Rural/Remote GP Training and Practice

The two major themes that emerged from the first set of interviews were divided into attractors for GP training in remote areas, and barriers to rural/remote GP training and practice. Attractors were family and community lifestyle factors, individual intrinsic motivators and remote medicine experiences. The main themes for barriers highlighted work related, location or family factors, as shown in Fig. 2. Overall, participants listed more attractors than barriers to GP training and supervision in remote locations. However, whether factors were considered an attractor, or a barrier depended largely on the individual circumstances, suggesting that adequate preparation or matching of registrars and supervisors to their practice environments is an important factor.

#### Theme 1: attractors to GP training in rural and remote underserved areas

Attractors had sub-themes of family and community lifestyle factors, individual motivators, and remote medicine experiences. The most frequently reported attractors were the community lifestyle and family benefits. A supervisor noted that *“being a young family in a town like this is really important because it allows you to be absorbed into the community”* (Supervisor, S2I). Another prominent attraction was the experience provided through practising remote medicine, in particular the importance of a good reputation for teaching and supervision for attracting registrars. For instance, a supervisor explained what attracted them to rural medicine *“rural medicine offers variety and highly rewarding work”* (Supervisor, S1F). Similarly, a registrar noted that *“being able to have access to good supervision both here and in the general practice and at the hospital is so important – it is the reason I came out”* (Registrar, R1E). This comment implies that a reputation for providing excellent educational and clinical experiences might be a major attractor and retention factor for GP training, especially in remote locations. Overall, important individual intrinsic motivators were prior rural experience, a rural background, and a passion for remote medicine and remote training requirements, highlighting the effectiveness of current strategies such as localised support from supervisors and JCU GPT. These strategies provided excellent learning opportunities and experience. See Table 2 below for further description and examples of the themes.

**Table 2:** Attractors for GP training in rural and remote areas

Sub-Themes	Description	Representative Quotes
Community Lifestyle and Family	The attractions of a rural lifestyle, being part of a community, importance of services and opportunities for families were the most frequent responses.	*“You are a part of a community, you have to look after these people, all the way through good times and in bad times.”* (Supervisor, S1I) *“Being able to get home and have dinner with their family.”* (Manager, K1I)
Remote Medicine Experiences	The scope of practice and variety of patient presentations, the workload flexibility, the quality of the supervision and opportunities for career advancement were all seen as important attractors for participants.	*“Your job is very diverse. You go from general practice to emergency through to theatre. Every day is completely different. It is fun, it’s interesting.”* (Registrar, R1I) *“It’s the variety of things we see and how quickly your confidence improves as well.”* (Registrar, R1G)
Individual Motivators	Prior rural experience and a desire to work rurally were reported as motivators as were rural training requirements	*“I was not thinking about it but I had rural rotation when I was working at Royal Brisbane and I came out here. I enjoyed it so I came back the next year.”* (Registrar, RIJ) *“I was born and grew up rural – that is what identifies me.”* (Supervisor, S3I)

#### Theme 2: barriers to GP training in rural and remote underserved areas

Barriers included sub-themes of workplace challenges, family needs and geographical isolation. An important barrier was described by a supervisor *“that expectation and stress without having the skillset and training is really difficult”* (Supervisor, S3G). This statement highlights the importance of good preparation and support for rural and remote practice. Family needs referred to the availability of schooling and activities for children, distance to family and friends and lack of opportunities for partners. This is exemplified in the following comment from a manager who said *“a lot of [doctors] will not stay if the kids are going into high school or schooling”* (Practice Manager, K1B). Geographical isolation was exacerbated by the availability of community services, travel distances and the associated costs. For example, one participant noted that *“it is far away from everywhere, so you have got to add a whole day for travel just because of the time of flights and the cost”* (Registrar, R1E). See Table 3 below for further description and examples of the themes.

**Table 3:** Barriers to remote GP training and practice

Sub-Themes	Description	Representative Quotes
Workplace Challenges	The challenging work environment and accessibility of additional educational opportunities and services are seen as deterrents and career limiting by some.	*“The hours and the on-calls can be very challenging.”* (Registrar, R1E) *“Lack of services is a big issue. Having to refer someone for something as basic as an ultrasound - it’s a 2-hour drive for patients to do that.”* (Manager, K1J) *“That expectation and stress without having the skillset and training is really difficult.”* (Supervisor, S3G) *“Access to education sometimes can be a bit frustrating but you try to make up for it and plan ahead.”* (Registrar, R3G)
Family Needs	Limited schooling, extra-curricular activities, distance from family and friends and opportunities for partners were also perceived barriers	*“The biggest impact for us at the moment is that we have two teenage sons who are both high school age and are both at boarding school.”* (Supervisor, S1F) *“The job prospects for partners, that’s the big issue.”* (Registrar, R2I)
Geographical Isolation	Limited community services and the isolation were considered barriers.	*“The cultural stuff and things that you miss out on a bit here.”* (Registrar, R3G) *“The professional and personal isolation is not great, and part of the reason why I enjoy getting to the city.”* (Supervisor, S4I)

#### Phase 2 findings

Findings from the second set of interviews and focus group broadly reported one main theme regarding participants’ perspectives of the training program on both GP registrars and supervisors. This theme represents the impacts of localised training on the GP workforce. Overall, participants reported that the training program had more positive than negative impacts on GP registrars and supervisors.

#### Theme 1: impacts of localised training on the GP workforce

**Fig. 3:**
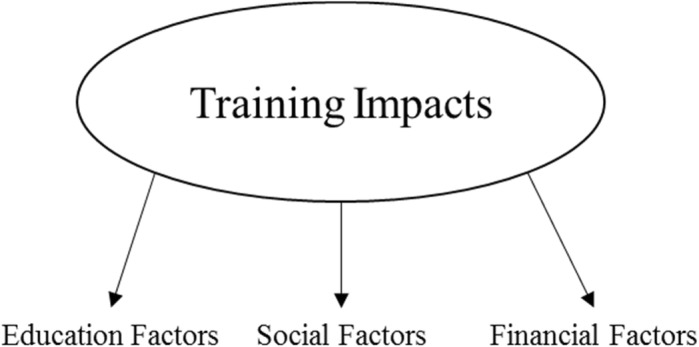
Factors Impacted by Rural/Remote GP Training

Participants reported that localised training impacted registrars through educational, social and financial factors. For supervisors, the perception of increased support as a result of the training program (e.g., administration, recruitment) was a predominant subtheme. The main theme and subthemes are presented in Fig. 3 and further explored in the sections below.

##### Education factors

The most reported impacts on the registrars resulting from the training program related to their improved knowledge and skills. Predominant themes within education included access to training, breadth of training, rural-specific knowledge, quality of training and the rewarding experience. Some participants reported a perceived positive influence on registrars’ mental health. This influence was specifically in terms of positive work-home spillover. For example, one participants noted *“…in the workforce if that person is quite happy and feels like they are well supervised and they are getting the training that they require, and then they are getting the support that they require, then I suppose their family would…benefit from that because that person is going to be quite happy in the way that they work”* (Community 5). Further description and examples of these themes are presented in Table 4 below.

**Table 4:** Education factors

Sub-Themes	Description	Representative quotes
Access to Training	Improves registrars’ ability to access training.	*“To be able to stay in their town that they live and they work in, and get the training that their city counterparts do, and to be able to provide that, I think that’s equitable and fair.”* (Community 5) *“Over the years we have had people who they’ve trained…and then come as registrars. So I think there is that sort of continuum of…students and then registrars.”* (Supervisor 5)
Breadth of Training	Broad scope of practice and range of experiences provided by the training.	*“The registrar training positions are often dual positions, so there’ll be a GP registrar and also a medical officer with their own private practice in the hospital. So, they actually hold two positions, which is quite attractive.”* (Community 4) *“I think one of the strengths of the set up here is the combination of hospital and that they do primary care as well.”* (Supervisor 5)
Rural Specific Knowledge	As a result of the training, registrars were perceived to have a greater depth of knowledge and understanding of the communities in which they practice.	*“We have people who are coming through who are aware…of the gap for the underprivileged parts of our society…It is a factor of both…the disparity between European and Aboriginal and Torres Strait Islander folks but also the element of isolation.”* (Manager 3) *“The orientation that the registrars are given when they come back probably have a better focus or preparing them for being in a remote area.”* (Supervisor 6)
Quality of Training and Supervision	Registrars completing the program were reported to receive quality training and supervision.	*“Already they come in with a greater level of confidence of their own experience of the training program.”* (Supervisor 2) *“The quality is really good, because they get the right direction.”* (Manager 3)
Rewarding Experience	Being involved with the training was reported to be a rewarding experience for the registrars and provides registrars the experience of living and working in rural communities.	*“There are health impacts on our community and other small communities that might just go under the radar in their training in larger hospitals.”* (Community 20) *“Well, I think it is a good thing in the fact that it gives these young doctors the experience of what it’s like to live and work in these small rural towns.”* (Community 3)

##### Social factors

Participants noted the improved relationships between registrars and the community, as well as within the profession, as a result of the localised training. For example, a supervisor noted *“I think the availability of the local node more relates to having someone on the ground who understands what the needs are in the community”* (Supervisor 6). Further description and examples of these themes are presented in Table 5 below.

**Table 5:** Social factors

Sub-Themes	Description	Representative quotes
Relationships with Community	Registrars involved with the training were reported to be more willing to engage with the community outside of work due to a greater understanding of rural communities.	*“There seems to be less reluctance to be involved in sporting or community organisations and groups.”* (Community 8) *Registrars become invested in local life and committed to patients.* (Manager 1)
Relationships with Profession	The localised presence of training was reported to improve networks and support for registrars and supervisors.	*“Every registrar that comes in will have some relationship with a hospital…Many of these general practice settings don’t have a relationship with a hospital…So they can be a good link and build some of that connectivity and relationship stuff.”* (Manager 4) *“We saw that far greater support coming from the training provider for remote, rural registrars and for the idea of having rural and remote registrars.”* (Supervisor 7)

##### Financial factors

Participants reported improved funding for training and increased preference to hire registrars trained through this program. The perception of improved funding available for GPs is reflected in one participant’s report: *“…it has a huge impact having that funding available for GPs in rural and remote areas. Without it, I just do not think that those new GPs and GP registrars…would feel very supported”* (Community 5). A supervisor also reported an increased preference for hiring registrars through the program when stating: *“…we would more likely take a candidate who had the JCU general practice training, because we know what will be their level of skills and expertise, of the graduates we have seen so far”* (Supervisor 2).

## Discussion

The current study has provided a contemporary overview of the issues encountered in expanding GP training capacity in rural and remote underserved communities to improve the alignment of training opportunities with community and workforce needs. The findings supported previous research around prior rural experience, an interest in remote medicine, and rural background as attractors.

While many of the identified issues are similar to the challenges in recruitment and retention reported previously [3–6, 8, 12–14], some new issues have emerged, perhaps because this study targeted training in smaller remote communities. A wide and varied scope of practice and its impact in attracting and retaining registrars and supervisors to remote areas was important. In addition, the quality of the workplace (administration as well as leadership), clinical practice, and clinical supervision is emerging as a key issue for attraction and retention. Registrars may accept being located in a smaller community for a period of time, but want to know they will have equivalent learning opportunities, access to learning resources and good quality supervision. Registrars with procedural interests and skills are concerned that these opportunities may be less available in smaller communities, where hospitals (if present) are smaller. Strategies for maintaining these skills include regular, planned opportunities for upskilling and continuing professional development in concert with larger rural hospitals in the region (which may appreciate the additional skills mix), and the local referral hospital. In accordance, Smith and colleagues propose eight key features of remote medical practice, arguing that “preparation for remote medical practice and the maintenance of professional standards poses unique challenges in the remote context”. This, in turn, reasons for a specific curriculum and training program for remote practice, with appropriate specific professional development [20].

Furthermore, the current study explored the impact that localised training (rural and remote workplace based training and local support) has on both GP registrars and supervisors within rural and remote areas. The localised training program was reported to mitigate the previously identified issues associated with rural and remote practice such as difficulty accessing professional development, and social and professional isolation [7, 8]. Indeed, it was identified that the localised training was perceived to facilitate building community and professional networks. The provision of professional and social support through the localised training program may assist in improving the recruitment of doctors to these underserved areas [11]. The current study supports the view that localised training programs can promote the attractors and lessen the barriers associated with rural and remote practice. The intrinsic richness of GP vocational training in rural areas identified in this study has also been reported in other studies [14, 21]. Nonetheless, the localised GP training model described in this study can be distinguished from other approaches to rural/remote training particularly because of the unique emphasis on individualised support provided to registrars by a strong, efficient network of community-based infrastructure, expertise and resources, to help map their training pathway and review progress at regular intervals. Furthermore, localised training can also support transformation of rural hospitals into teaching health services. An example is one North Queensland report which noted the importance of local commitment to quality health services, leadership, local coordination, community support and links between key organizations, concluding that “as both clinical and teaching capacity develops, the workforce may stabilise, infrastructure and teaching culture are established, and long-term recruitment and retention strategies emerge” [6].

### Limitations

Although the current study is limited to a single state and the footprint of only one postgraduate training organisation, the JCU GPT program covers a large and diverse region that hosts training for a substantial proportion of rural and remote doctors in Australia and findings may be relevant to other training regions. The diversity may itself be both a strength and a limitation. However, the range of responses and data saturation of interviews suggests confidence in the accuracy and interpretation of findings.

## Conclusion

JCU GPT has implemented a strategy of expanding training opportunities in smaller rural and more remote, mostly inland communities as a means of increasing rural and remote medical workforce. Results have shown that, while some factors may have minimally changed over the last 20 years with respect to recruitment and retention challenges, some new issues specific to training have emerged. Strategies to address these challenges include enhancing clinical and educational experiences of working in more remote communities by matching scope of practice to registrar interests to improve their clinical and educational opportunities. Also, a reputation for providing excellent educational and clinical experiences emerged as a new key factor influencing attraction and retention and might become an effective strategy for workforce recruitment to health services in remote underserviced areas. These strategies may be effectively applied through a localised training program that prepares future GPs in the context in which they will continue practice.

## Data Availability

The interview and focus group protocol and datasets produced and/or analysed during the current study are available from the corresponding author on reasonable request.

## Abbreviations

GP: General Practitioner
IA: Index of Access
JCU GPT: James Cook University General Practice Training
MMM: Modified Monash Model
